# Identification of Crucial Genes Associated With Immune Cell Infiltration in Hepatocellular Carcinoma by Weighted Gene Co-expression Network Analysis

**DOI:** 10.3389/fgene.2020.00342

**Published:** 2020-04-24

**Authors:** Dengchuan Wang, Jun Liu, Shengshuo Liu, Wenli Li

**Affiliations:** ^1^Office of Medical Ethics, Shenzhen Longhua District Central Hospital, Shenzhen, China; ^2^Departments of Clinical Laboratory, Yue Bei People’s Hospital, Shantou University Medical College, Shaoguan, China; ^3^School of Pharmacy, Henan University, Kaifeng, China; ^4^Reproductive Medicine Center, Yue Bei People’s Hospital, Shantou University Medical College, Shaoguan, China

**Keywords:** weighted gene co-expression network analysis, hepatocellular carcinoma, immune infiltrate, key gene, TCGA

## Abstract

The dreadful prognosis of hepatocellular carcinoma (HCC) is primarily due to the low early diagnosis rate, rapid progression, and high recurrence rate. Valuable prognostic biomarkers are urgently needed for HCC. In this study, microarray data were downloaded from GSE14520, GSE22058, International Cancer Genome Consortium (ICGC), and The Cancer Genome Atlas (TCGA). Differentially expressed genes (DEGs) were identified among GSE14520, GSE22058, and ICGC databases. Weighted gene co-expression network analysis (WGCNA) was used to establish gene co-expression modules of DEGs, and genes of key modules were examined to identify hub genes using univariate Cox regression in the ICGC cohort. Expression levels and time-dependent receiver operating characteristic (ROC) and area under the curve (AUC) were determined to estimate the prognostic competence of the hub genes. These hub genes were also validated in the Gene Expression Profiling Interactive Analysis (GEPIA) and TCGA databases. TIMER algorithm and GSCALite database were applied to analyze the association of the hub genes with immunocytotic infiltration and their pathway enrichment. Altogether, 276 DEGs were identified and WGCNA described a unique and significantly DEGs-associated co-expression module containing 148 genes, with 10 hub genes selected by univariate Cox regression in the ICGC cohort (BIRC5, FOXM1, CENPA, KIF4A, DTYMK, PRC1, IGF2BP3, KIF2C, TRIP13, and TPX2). Most of the genes were validated in the GEPIA databases, except IGF2BP3. The results of multivariate Cox regression analysis indicated that the abovementioned hub genes are all independent predictors of HCC. The 10 genes were also confirmed to be associated with immune cell infiltration using the TIMER algorithm. Moreover, four-gene signature was developed, including BIRC5, CENPA, FOXM1, DTYMK. These hub genes and the model demonstrated a strong prognostic capability and are likely to be a therapeutic target for HCC. Moreover, the association of these genes with immune cell infiltration improves our understanding of the occurrence and development of HCC.

## Introduction

Hepatocellular carcinoma (HCC) is a fatal tumor with a poor prognosis due to the broad range of its underlying systemic symptoms. Epidemiology reports have ranked HCC as the third leading cause of cancer death globally for years. The incidence of HCC is increasing in regions that have conventionally been low incidence areas, such as North America and some European countries (Kulik and El-Serag, 2019). With the development of diagnostic techniques, HCC is increasingly being diagnosed at an early stage. However, due to its high recurrence rate, rapid progression, and short overall survival (OS) time, the prognosis of patients with HCC is not satisfactory (Bruix et al., 2014; Zheng et al., 2017). Therefore, it is necessary to screen and identify new prognostic markers for HCC.

Alpha-fetoprotein (AFP) and AFP mRNA have been used as potential prognosis biomarkers for HCC (Hanazaki et al., 2001). However, since they rely on significant tumor burden, their applications have certain limitations, and the evaluation of their value has been incomplete (Tangkijvanich et al., 2000). As a result, it is important to identify new diagnostic and prognostic markers. Bioinformatics analysis has been widely used for screening molecules (e.g., functional genes, micro-RNAs, and long non-coding RNAs) that contribute toward disease progression, treatment response, and prognosis (Villa et al., 2016; Li et al., 2019; Unfried et al., 2019). Immune-related gene may be an important prognostic factor for HCC (Xie et al., 2018). Upregulated expression of LINC00978 is a marker of poor prognosis in HCC (Xu X. et al., 2019). In addition, elevated expression of TXNDC12 has been correlated with elevated expression of nuclear β-catenin and with OS and disease-free survival (Yuan et al., 2019). These studies indicated that next-generation sequencing could be performed to distinguish the biomarkers of HCC. Likewise, we selected the prognosis genes and signature using high-throughput sequencing.

In the present study, we screened differentially expressed genes (DEGs) from the Gene Expression Omnibus (GEO) and International Cancer Genome Consortium (ICGC) datasets. We also used weighted gene co-expression network analysis (WGCNA) to identify the association between gene expression modules and clinical features. The top 10 genes were screened out using univariate Cox regression analysis. These genes were verified in the Gene Expression Profiling Interactive Analysis (GEPIA) and The Cancer Genome Atlas (TCGA) databases. The 10 hub genes identified by bioinformatics were upregulated in HCC and able to predict prognosis, thus providing highly reliable analytic results.

## Materials and Methods

### Data Acquisition

Messenger RNA (mRNA) expression and corresponding clinical information (Table 1) for HCC patients were obtained from the GEO database^1^, ICGC database^2^, and TCGA database^3^.

**TABLE 1:** Information of HCC patients in TCGA and the ICGC.

**Clinical characteristics**		**Total**	**%**	**Clinical characteristics**		**Total**	**%**
**TCGA**		370				370	
Survival status	Survival	244	65.95	T	T1	181	48.92
	Death	126	34.05		T2	93	25.14
Age	≤65 years	232	62.7		T3	80	21.62
	>65 years	138	37.3		T4	13	3.51
Grade	G1	55	14.86	M	M0	266	71.89
	G2	177	47.84		M1	4	1.08
	G3	121	32.7		MX	100	27.03
	G4	12	3.24	N	N0	252	68.11
Stage	I	171	46.22		N1	4	1.08
	II	85	22.97		NX	113	30.54
	III	85	22.97	Gender	Male	249	67.3
	IV	5	1.35		Female	121	32.7
**ICGC**		232				232	
Survival status	Survival	189	81.47	Stage	I	36	15.52
	Death	43	18.53		II	106	45.69
Age	≤65 years	90	38.79		III	71	30.6
	>65 years	142	61.21		IV	19	8.19
Gender	Male	171	73.71	Prior malignancy	No	202	87.07
	Female	61	26.29		Yes	30	12.93

*HCC, hepatocellular carcinoma; ICGC, International Cancer Genome Consortium; TCGA, The Cancer Genome Atlas.*

### Data Preprocessing and Analysis of Differentially Expressed Genes

The GSE14520 and GSE22058 datasets were collected from the GEO dataset. GSE14520 (GPL3921, Affymetrix HT Human Genome U133A Array) includes 220 normal and 225 tumor tissues. GSE22058 (GPL6793, Human RSTA Custom Affymetrix 1.0 microarray) contains 97 normal and 100 tumor tissues. The ICGC-LIRI profiles that were downloaded included 202 normal and 243 tumor tissues. The validation dataset with mRNA expression profile and clinical information was downloaded from TCGA. Preprocessing of the downloaded raw data included background adjustment, normalization, and gene biotype re-annotation. DEGs between tumor and adjacent tissues were identified using the R package “limma.” Absolute log2 fold-change >1 and *P* < 0.05 were considered statistically significant. The overlapping DEGs were portrayed using a Venn diagram^4^.

### Construction of Co-expression Gene Networks

Weighted gene co-expression network analysis was performed as previously described to describe the correlation patterns among genes (Langfelder and Horvath, 2008). Expression profile data of DEGs and phenotypic data matrix in ICGC were obtained. The data comprised a total of 232 samples, 276 genes, and five phenotypes. Genes expressing NA were removed. All the samples were analyzed, and outliers in the clustering results were eliminated. The revised data expression profile included 232 samples and 264 genes.

### Functional Annotation and Pathway Enrichment Analysis

Gene Ontology (GO) and Kyoto Encyclopedia of Genes and Genomes (KEGG^5^) analyses were performed using the “ClusterProfiler package” in R for functional annotation and pathway enrichment, respectively. The pathway enrichment analysis of hub genes was done using GSCAlite^6^, a web-based analysis platform for analysis of cancer genes (Liu et al., 2018).

### Hub Gene Screening and Validation

Prognostic genes were distinguished in ICGC cohorts by univariate Cox regression using a cutoff of *P* < 0.05. Among the prognostic genes, the top 10 genes with low *P*-values were identified as hub genes. Kaplan–Meier survival curve and the time-dependent receiver operating characteristic (ROC) curve were constructed to assess the predictive potential of these genes using the “survival” and “survivalROC” functions of the R package. Survival curves for the HCC patients were plotted using data from TCGA and the GTEx-based GEPIA database^7^ (Tang et al., 2017) to confirm the genes contributing to survival. These highly expressed genes in HCC patients had been corroborated beforehand using the GEPIA database. Finally, univariate and multivariate Cox regression analyses were performed in TCGA datasets to assess whether these hub genes could be independent predictors along with other clinicopathological features for HCC patients. UALCAN database^8^ and Cbioportal^9^ database were used to assess methylation and mutation of the hub genes particularly.

### Tumor-Infiltrating Immune Cells

The Tumor Immune Estimation Resource (TIMER) database^10^ uses RNA-seq expression profile data to detect the infiltration of immune cells in tumor tissues and assess the hub genes relationship with the immune cells (Li et al., 2017). This strategy was followed in this study.

### Construction of Prognostic Model and Nomogram

In order to find the most relevant prognostic genes, the hub genes were performed to construct prognostic risk signature using multivariate Cox regression in ICGC database. We applied a stepwise method to further identify the best model. Then, four-gene signature including CENPA, DTYMK, BIRC5, FOXM1 were settled and Prognostic index (Pi) = (β ^∗^ expression level of CENPA) + (β ^∗^ expression level of DTYMK) + (β ^∗^ expression level of BIRC5) + (β ^∗^ expression level of FOXM1). The prognostic value of the model was examined through Kaplan–Meier survival curve and the time-dependent ROC curve in the training set of ICGC and the testing set of TCGA. Subsequently, univariate and multivariate Cox regression analyses were used to evaluate whether the four-gene signature could be an independent prognostic factor with other clinical information, including age, sex, stage, tissue registration, and T staging. Finally, we constructed a nomogram based on the independent clinical prognostic factor to estimate the expectation of 1, 3, and 5 years in HCC.

## Results

### Identification of Differentially Expressed Genes

**FIGURE 1:**
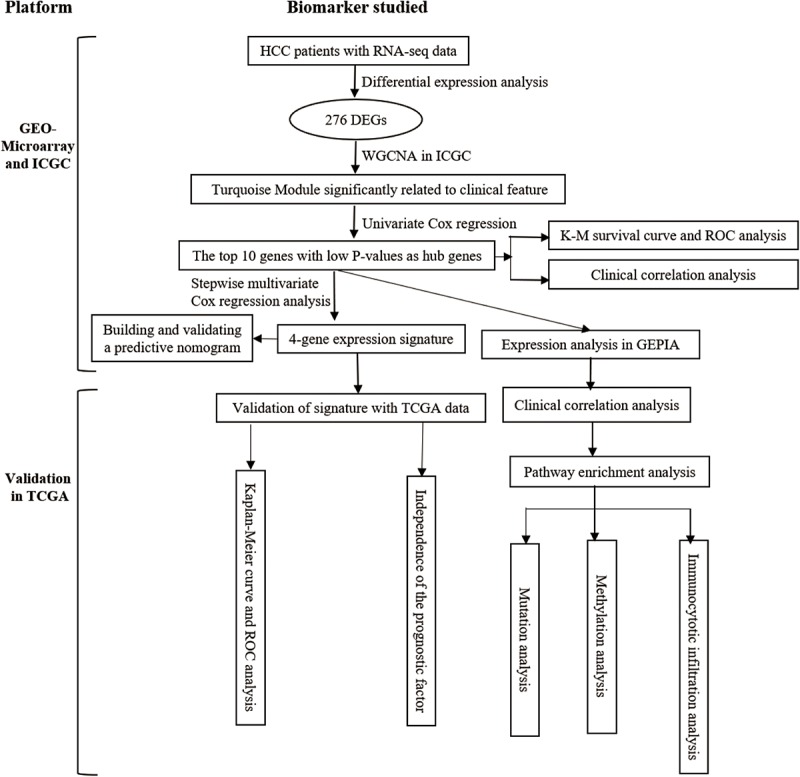
Overall flowchart of this study.

**FIGURE 2:**
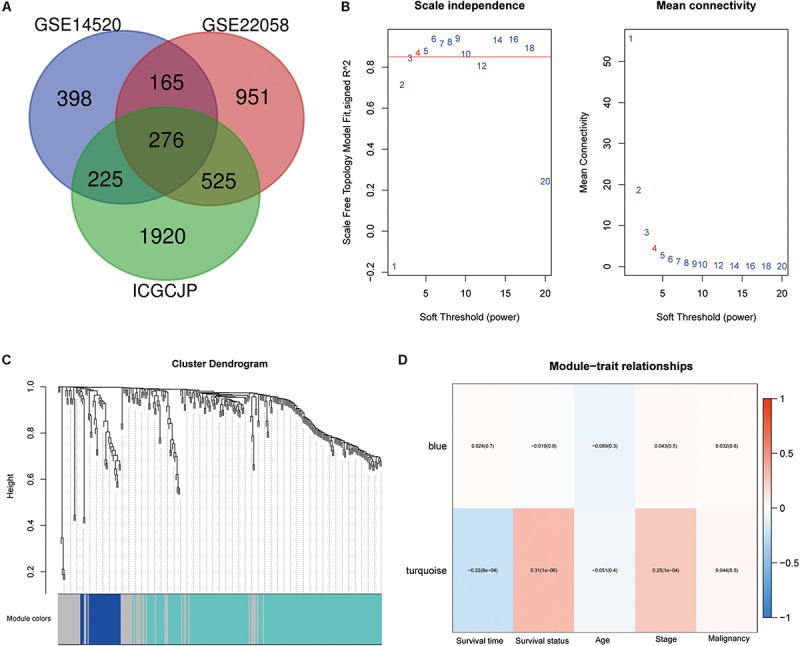
Differentially expressed genes (DEGs) identification and weighted gene co-expression network analysis (WGCNA) construction. **(A)** Venn diagram analysis of DEGs between GSE14520, GSE22058, and ICGCJP. **(B)** Identification of the soft threshold according to the standard of the scale-free network. **(C)** Identification of co-expression modules in hepatocellular carcinoma (HCC). **(D)** Correlation between gene modules and clinical traits.

The whole work of this study is shown in Figure 1. The DEGs of mRNA expression profiles, including GSE14520, GSE22058, and ICGC datasets, were shown in the volcano map (Supplementary Figure S1). A total of 276 DEGs were recognized in HCC tissues compared with non-cancerous tissues. The DEGs comprised 138 upregulated genes and 138 downregulated genes (Figure 2A). Gene co-expression modules for the expression of DEGs were established in the ICGC cohort using WGCNA. The co-expression network was consistent with the scale-free network. The logarithmic value log (k) of the node with connectivity k was negatively correlated with the logarithmic log [p (k)] of the probability of the node, and the correlation coefficient was >0.8. We chose the soft threshold of β = 4 to ensure that the network was scale-free (Figure 2B). Based on the hybrid dynamic shearing tree standard, the minimum number of genes was set at 30 per gene network module. In the total of three modules shown in Figure 2C, the gray module is a set of genes that could not be aggregated into other modules. Gene statistics in each module are presented in Table 2. We calculated the correlation between these modules and each phenotype according to the eigenvectors of each module. The turquoise module denotes significant associations with the clinical features of HCC (Figure 2D).

**TABLE 2:** The gene numbers of each module.

**Module**	**Number**
Blue	31
Gray	55
Turquoise	187

### Functional Annotation and Pathway Enrichment Analysis

**FIGURE 3:**
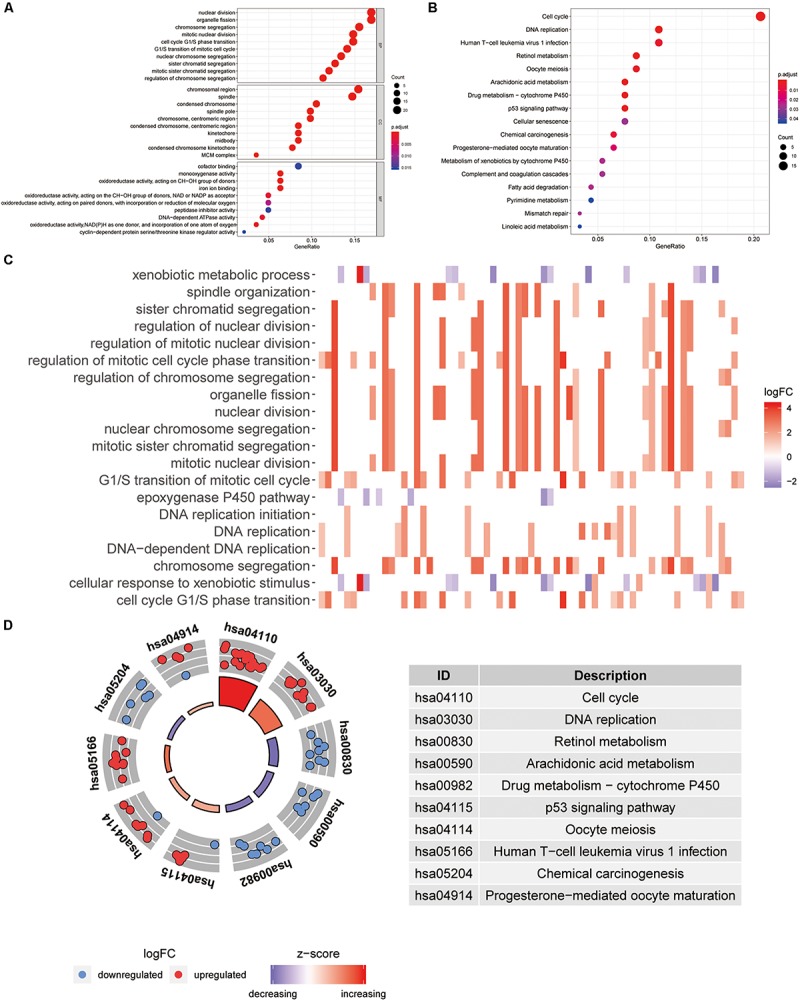
Functional annotation and pathway enrichment analysis. **(A)** Gene Ontology (GO) enrichment analysis. **(B)** Kyoto Encyclopedia of Genes and Genomes (KEGG) pathway enrichment analysis. **(C)** The heatmap of relationship between differentially expressed genes (DEGs) and GO terms. **(D)** The Z-score of enriched pathways.

All 148 common DEGs were analyzed by GO and KEGG pathway enrichment analyses. These data are presented as the turquoise module in Figure 1D GO analysis revealed three features. First, for biological processes (BPs), DEGs were particularly enriched in nuclear division, organelle fission, chromosome segregation, mitotic nuclear division, and so on. Second, for cell components (CCs), DEGs were significantly enriched for the chromosomal region, spindle, condensed chromosome and spindle pole, and so on. Third, for molecular functions (MFs), DEGs were enriched in cofactor binding, monooxygenase activity, oxidoreductase activity, acting on CH–OH group of donors, and iron ion binding, and so on (Figure 3A). A heatmap was constructed to show the relationships between DEGs and GO terms (Figure 3C). KEGG analysis demonstrated that DEGs were particularly enriched in the cell cycle, DNA replication, human T-cell leukemia virus 1 infection, p53 signaling pathway, and so on (Figure 3B). The Z-score of the enriched pathways indicated that cell cycle, DNA replication, progesterone-mediated oocyte maturation, human T-cell leukemia virus 1 infection, oocyte meiosis, and p53 signaling pathway were more likely to be increased (Figure 3D).

### Identification of Hub Genes

**FIGURE 4:**
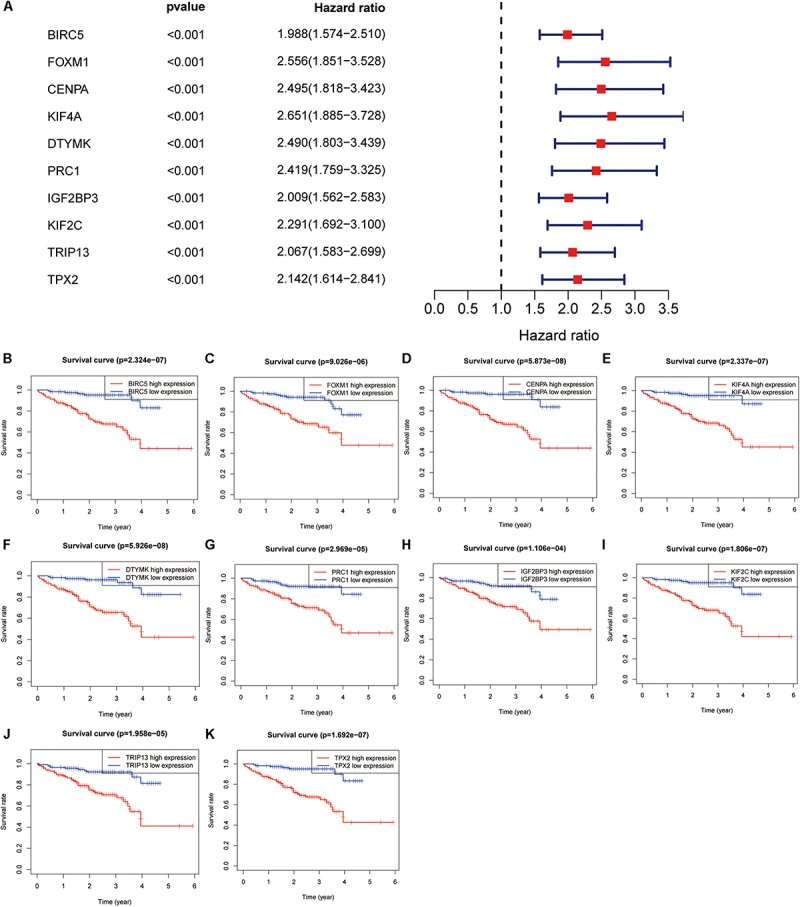
Hub genes identification in the International Cancer Genome Consortium (ICGC) dataset. **(A)** Top 10 genes with low *P*-value from prognostic genes. **(B–K)** Survival analysis of 10 genes for hepatocellular carcinoma (HCC) patients in the ICGC cohort, including BIRC5 **(B)**, FOXM1 **(C)**, CENPA **(D)**, KIF4A **(E)**, DTYMK **(F)**, PRC1 **(G)**, IGF2BP3 **(H)**, KIF2C **(I)**, TRIP13 **(J)**, and TPX2 **(K)**.

**FIGURE 5:**
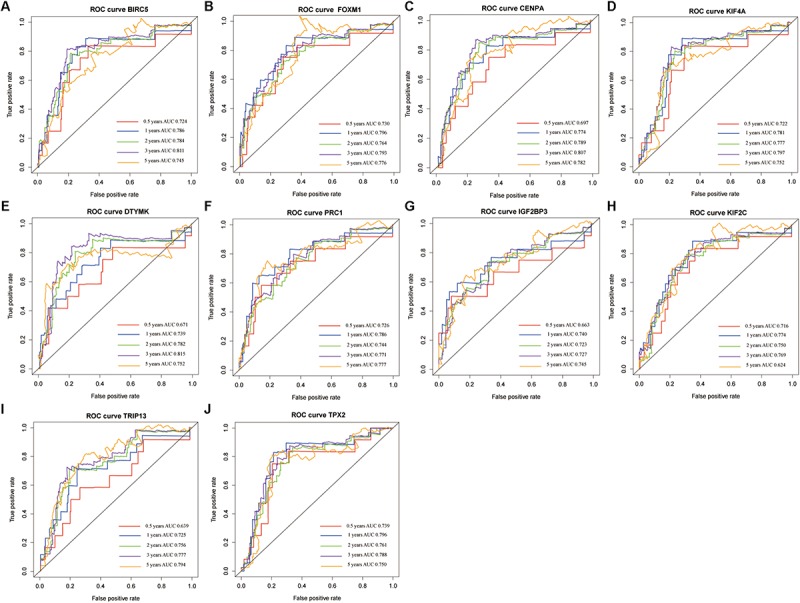
The time-dependent receiver operating characteristic (ROC) and area under the curve (AUC) of 10 hub genes in the International Cancer Genome Consortium (ICGC) dataset, including BIRC5 **(A)**, FOXM1 **(B)**, CENPA **(C)**, KIF4A **(D)**, DTYMK **(E)**, PRC1 **(F)**, IGF2BP3 **(G)**, KIF2C **(H)**, TRIP13 **(I)**, and TPX2 **(J)**.

The genes in the turquoise module of the ICGC cohort were analyzed using univariate Cox regression to identify prognostic markers from among the survival-related candidates. Of the prognostic genes, the top 10 genes with low *P*-values were identified as hub genes (Figure 4A). The hub genes included baculoviral IAP repeat containing 5 (BIRC5), forkhead box M1 (FOXM1), centromere protein A (CENPA), kinesin family member 4A (KIF4A), deoxythymidylate kinase (DTYMK), protein regulator of cytokinesis 1 (PRC1), insulin like growth factor 2 mRNA binding protein 3 (IGF2BP3), kinesin family member 2C (KIF2C), thyroid hormone receptor interactor 13 (TRIP13), and TPX2 microtubule nucleation factor (TPX2). All 10 genes displayed strong prognostic correlations with HCC because of their high hazard ratios and low *P*-values. To evaluate the prognostic values of the 10 hub genes, survival curves for HCC patients in the ICGC cohort were plotted. The overexpression of all hub genes was significantly and negatively associated with the prognosis of the HCC patients (Figures 4B–K). According to the feature vectors of turquoise module, we calculated the correlation between the gene expression and the turquoise module (Supplementary Figure S2). Furthermore, the expression of these hub genes tended to be higher in patients with advanced clinical stages of HCC (Supplementary Figure S3). A time-dependent ROC curve was constructed, and the area under the curve (AUC) was calculated to estimate the prognostic competence of the hub genes (Figure 5). The AUC of the hub genes was >0.62, and their 3-year AUC was >0.70. The results indicated these genes have powerful predictive prognostic capacity.

### Validation of Hub Gene Expression and Survival Analysis Results

**FIGURE 6:**
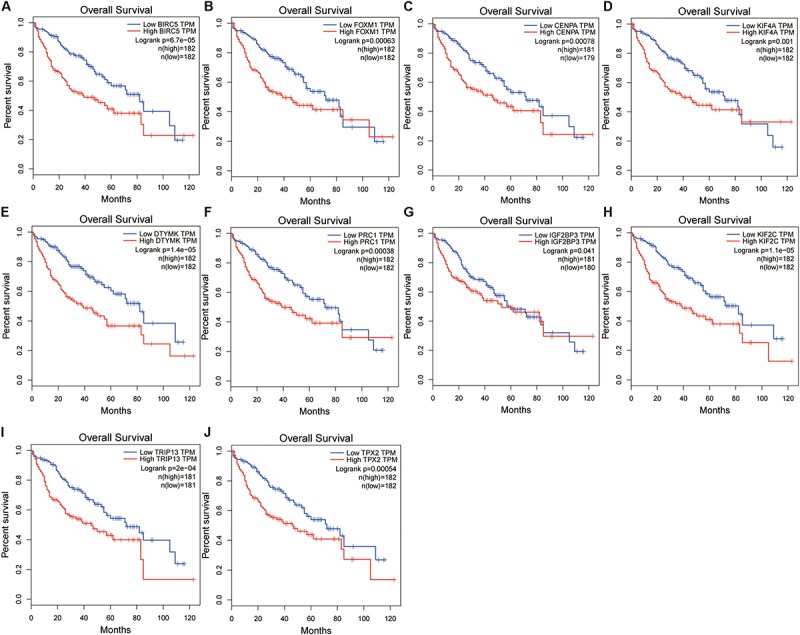
Validation of the hub gene expression levels in the Gene Expression Profiling Interactive Analysis (GEPIA) database, including BIRC5 **(A)**, FOXM1 **(B)**, CENPA **(C)**, KIF4A **(D)**, DTYMK **(E)**, PRC1 **(F)**, IGF2BP3 **(G)**, KIF2C **(H)**, TRIP13 **(I)**, and TPX2 **(J)**.

**FIGURE 7:**
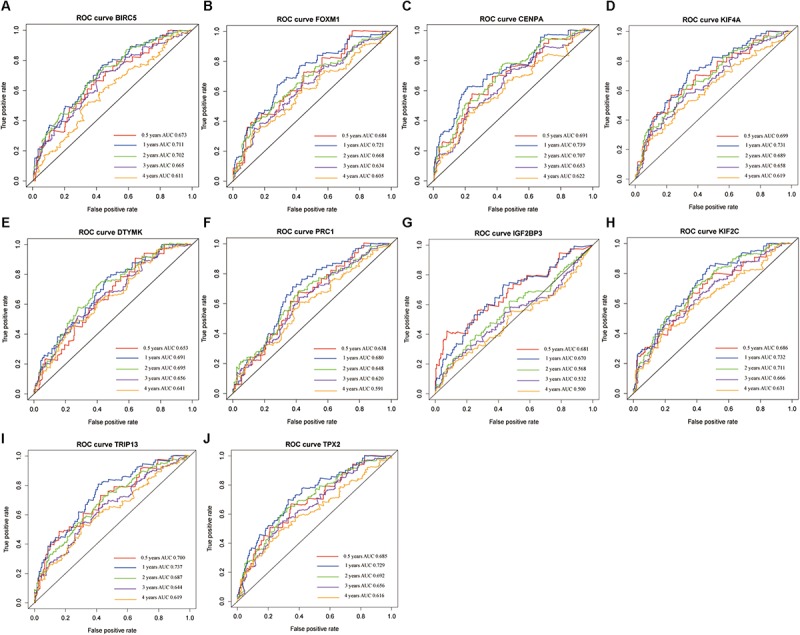
The time-dependent receiver operating characteristic (ROC) and area under the curve (AUC) of 10 hub genes in the Gene Expression Profiling Interactive Analysis (GEPIA) database, including BIRC5 **(A)**, FOXM1 **(B)**, CENPA **(C)**, KIF4A **(D)**, DTYMK **(E)**, PRC1 **(F)**, IGF2BP3 **(G)**, KIF2C **(H)**, TRIP13 **(I)**, and TPX2 **(J)**.

A confirmatory analysis was conducted using the GEPIA database to acquire more reliable analytic results. All hub genes, except IGF2BP3, were significantly overexpressed in HCC tissues (Supplementary Figure S4; *P* < 0.01). IGF2BP3 showed a tendency for high expression in tumors. Analysis of GEPIA data revealed that the expression levels of hub genes were significantly higher in Stage II and III than in Stage I HCC. Information concerning Stage IV was insufficient since there were only five Stage IV patients (Supplementary Figure S5). The survival analysis results of all hub genes were also validated in GEPIA databases. Overexpression of all hub genes consistently negatively predicted prognosis in patients with HCC, with the BIRC5, DTYMK, KIF2C, and TRIP13 genes having a greater prognostic value (Figure 6). The time-dependent ROC and AUC of hub genes also showed that these prognostic genes had high sensitivity and specificity (Figure 7), especially BIRC5, FOXM1, CENPA, KIF4A, KIF2C, TRIP13, and TPX2. The 1-year AUC of these genes were >0.70.

### Univariate and Multivariate Cox Regression Analyses of Hub Genes

**FIGURE 8:**
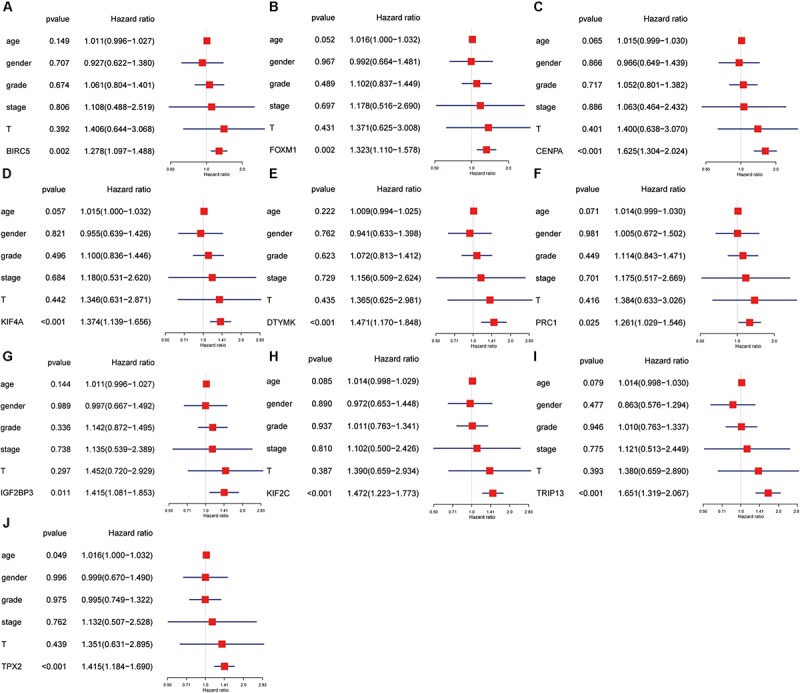
The univariate and multivariate Cox regression analysis of hub genes, including BIRC5 **(A)**, FOXM1 **(B)**, CENPA **(C)**, KIF4A **(D)**, DTYMK **(E)**, PRC1 **(F)**, IGF2BP3 **(G)**, KIF2C **(H)**, TRIP13 **(I)**, and TPX2 **(J)**.

Univariate and multivariate Cox regression analyses were performed to evaluate the independent predictive values of hub genes for HCC patients in TCGA cohort. The results of univariate Cox analysis indicated that all hub genes were prognostic factors, with the CENPA, DTYMK, IGF2BP3, KIF2C, and TRIP13 genes having higher hazard ratios (HRs) and lower *P*-values (Table 3). The results of multivariate Cox analysis further confirmed that all hub genes were independent prognostic factors associated with OS (Figure 8), especially CENPA (HR, 1.625; *P* < 0.001), KIF4A (HR, 1.374; *P* < 0.001), DTYMK (HR, 1.471; *P* < 0.001), KIF2C (HR, 1.472; *P* < 0.001), TRIP13 (HR, 1.651; *P* < 0.001), and TPX2 (HR, 1.415; *P* < 0.001).

**TABLE 3:** Univariate analysis of overall survival in TCGA.

**Parameters**	**HR**	**HR.95L**	**HR.95H**	***P*-value**
Age	1.010238	0.995394	1.025303	0.177451
Gender	0.82049	0.557003	1.208619	0.316744
Grade	1.120516	0.868279	1.446029	0.381849
Stage	1.671825	1.359423	2.056017	1.12E−06
T	1.651769	1.356646	2.011093	5.82E−07
BIRC5	1.390493	1.202188	1.608294	8.99E−06
FOXM1	1.388131	1.175026	1.639885	0.000115
CENPA	1.803812	1.471493	2.21118	1.36E−08
KIF4A	1.461444	1.227241	1.740342	2.06E−05
DTYMK	1.68016	1.3456	2.097902	4.65E−06
PRC1	1.355446	1.122636	1.636537	0.001561
IGF2BP3	1.576372	1.212821	2.048901	0.000668
KIF2C	1.600898	1.343936	1.906991	1.35E−07
TRIP13	1.726845	1.415869	2.106122	6.94E−08
TPX2	1.48561	1.265967	1.74336	1.24E−06

*HR, hazard ratio; TCGA, The Cancer Genome Atlas.*

### Immunocytotic Infiltration, Methylation, Mutation, and Pathway Enrichment Analyses

**FIGURE 9:**
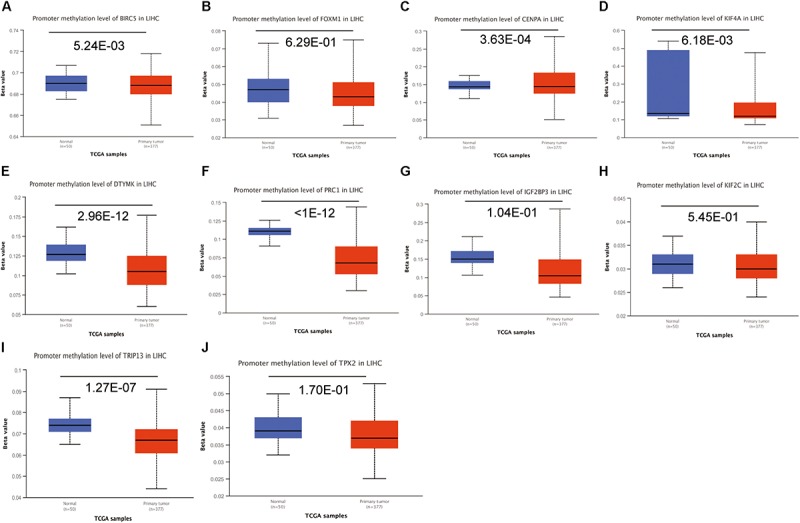
The analysis of DNA methylation. Analysis of the hub genes methylation in tumor and normal tissues, including BIRC5 **(A)**, FOXM1 **(B)**, CENPA **(C)**, KIF4A **(D)**, DTYMK **(E)**, PRC1 **(F)**, IGF2BP3 **(G)**, KIF2C **(H)**, TRIP13 **(I)**, and TPX2 **(J)**.

**FIGURE 10:**
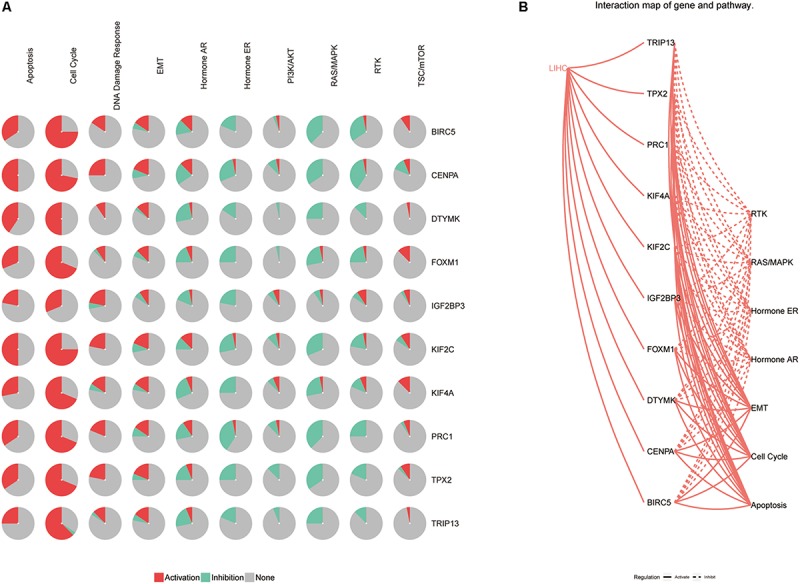
The pathway enrichment analysis of hub genes. **(A)** The relationship between pathways and hub genes. **(B)** The interaction between pathways and hub genes.

To investigate the potential mechanism of hub genes in HCC, the TIMER algorithm and GSCALite database were applied to analyze the immunocytotic infiltration and pathway enrichment. TIMER algorithm analysis revealed a correlation between hub gene expression levels and immunocytotic infiltration. The expression levels of the BIRC5, FOXM1, CENPA, KIF4A, PRC1, KIF2C, and TPX2 genes were strongly associated with abundant infiltration of CD4^+^ T cells, CD8^+^ T cells, B cells, macrophages, neutrophils, and dendritic cells in HCC (Supplementary Figures S6, S7). The immunocytotic infiltration analysis revealed that the hub gene expression levels were significantly correlated with most immune marker sets of various immune cells, including different T cells, in HCC. DNA methylation plays crucial roles in tumorigenesis. Therefore, we investigated the difference of methylation between tumor and normal in TCGA. The results show that BIRC5, CENPA, KIF4A, DTYMK, PRC1, and TRIP13 have low beta values in tumor (Figure 9). The analysis of genetic mutation exposed that the percentage alteration in the mRNA expression levels of BIRC5, FOXM1, CENPA, KIF4A, DTYMK, PRC1, IGF2BP3, KIF2C, TRIP13, and TPX2 were 11%, 7%, 9%, 6%, 6%, 8%, 9%, 5%, 16%, and 11%, separately (Supplementary Figure S8). Pathway enrichment analysis of hub genes indicated that apoptosis, cell cycle, and epithelial–mesenchymal transition (EMT) pathway were activated, and hormone androgen receptor (AR), hormone estrogen receptor (ER), RAS/mitogen-activated protein kinase (RAS/MAPK), and receptor tyrosine kinase (RTK) were inhibited in HCC (Figure 10). Many studies have demonstrated the participation of the cell cycle, apoptosis, and EMT pathway in the development of cancer. Therefore, the hub genes may be important for the malignant progression of HCC.

### Constructions and Validation of the Four-Gene Signature

**FIGURE 11:**
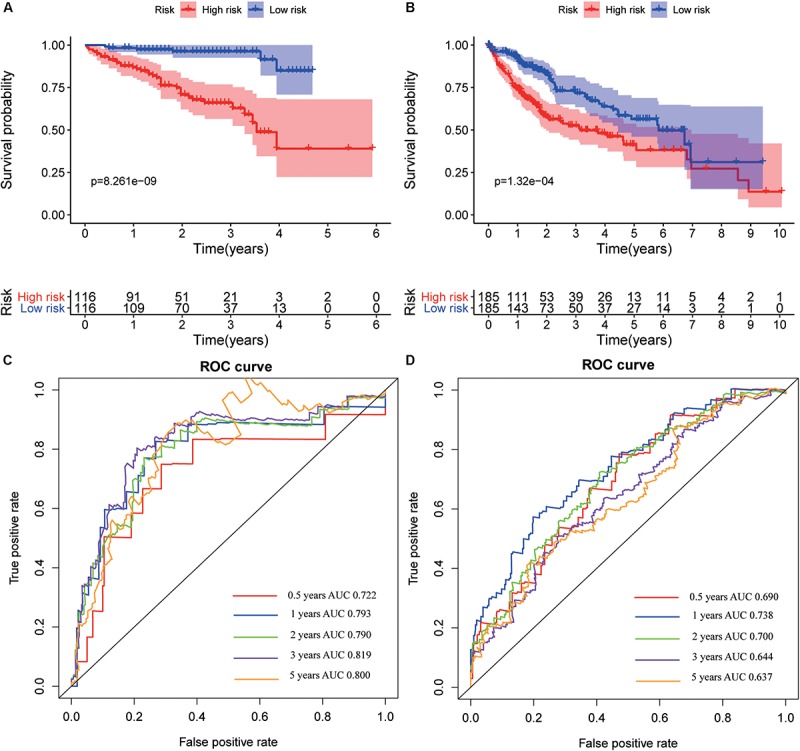
Kaplan–Meier survival and receiver operating characteristic (ROC) curves in training and validation datasets. Kaplan–Meier survival curves of overall survival for hepatocellular varcinoma (HCC) patients in the International Cancer Genome Consortium (ICGC) **(A)** and The Cancer Genome Atlas (TCGA) **(B)** set. ROC curves evaluate the predictive power for 0.5, 1, 2, 3, and 5 years in the ICGC **(C)** and The Cancer Genome Atlas (TCGA) **(D)**.

The hub genes were applied to construct a prognostic model using multivariate Cox regression in ICGC database. Next, we built a four-gene signature, and the risk score = (0.26 ^∗^ expression level of CENPA) + (0.23 ^∗^ expression level of DTYMK) + (0.06^∗^ expression level of BIRC5) + (0.46 ^∗^ expression level of FOXM1). Then, all patients were divided into low-risk group and high-risk group based on the median value of risk scores in the training set (ICGC cohort) and testing set (TCGA cohort). Comparing to the low-risk group, the high-risk group suffered from poorer progression and higher expression of mRNA (Supplementary Figure S9). Subsequently, the analysis of the K-M curve indicated that the low-risk group presents a favorable outcome in training set (Figure 11A) and testing set (Figure 11B). Meanwhile, the AUCs were applied to assess the predictive power of the four-gene signature, and the larger the AUC, the better the model predictive capacity. The AUCs for 0.5-, 1-, 2-, 3-, and 5-year OS were 0.722, 0.793, 0.790, 0.819, and 0.800 in the training set (Figure 11C); 0.690, 0.738, 0.700, 0.644, and 0.637 in the testing set (Figure 11D), especially. Those results indicated that the model had an excellent performance for OS prediction.

### Independent Prognostic Factor and Nomogram Construction

**FIGURE 12:**
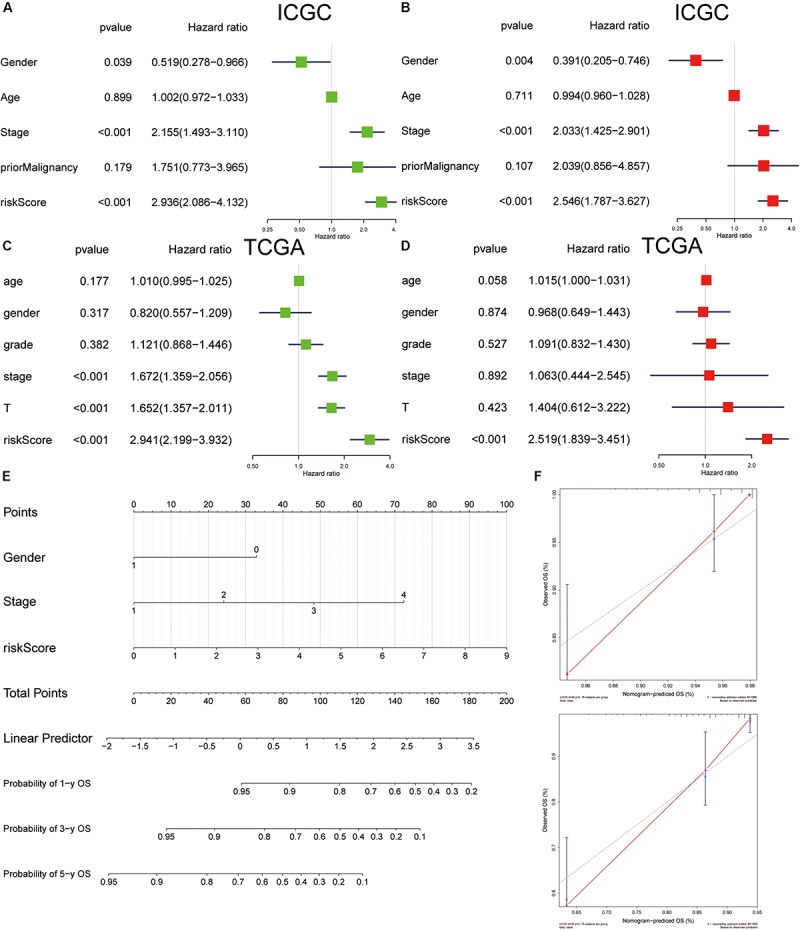
Cox regression analyses of clinical factors and construction of a nomogram for overall survival prediction in hepatocellular carcinoma (HCC). Univariate Cox regression analyses of clinicopathological factors for overall survival in the International Cancer Genome Consortium (ICGC) **(A)** and The Cancer Genome Atlas (TCGA) **(C)**. Multivariate Cox regression analyses of clinicopathological factors for overall survival in the ICGC **(B)** and TCGA **(D)**. **(E)** The nomogram consists of gender, stage, and risk score. **(F)** Calibration plot of the nomogram.

The analysis of univariate Cox regression revealed that gender (*P* = 0.039; HR, 0.519), stage (*P* < 0.001; HR, 2.155), and risk score (*P* < 0.001; HR, 2.936) in the ICGC cohort (Figure 12A), and stage (*P* < 0.001; HR, 1.672), T stage (*P* < 0.001; HR, 1.652), and risk score (*P* < 0.001; HR = 2.941) in TCGA cohort (Figure 12C) were associated with OS. Furthermore, multivariate Cox regression analysis supported that risk score (*P* < 0.001; HR = 2.546) was an independent prognostic facto in the ICGC cohort (Figure 12B), and the risk score (*P* < 0.001; HR, 2.519) was confirmed in TCGA (Figure 12D). Nomogram has been widely used for clinical evaluation; in this study, we developed a nomogram for predicting the OS in HCC patients based on risk score and clinical factor (Figure 12E). The calibration curve was applied to illustrate the consistence between estimation and actual probability (Figure 12F).

## Discussion

Hepatocellular carcinoma is a highly malignant tumor. It is often diagnosed at the mid or late stage of the disease (Forner et al., 2012). Surgery is still the most important approach for treating HCC; however, its therapeutic effect is not satisfactory (Bruix et al., 2014). New carcinoma biomarkers and therapeutic targets are needed. In this study, bioinformatics and comprehensive analyses of multiple datasets were used to screen 10 hub genes that proved to be independent prognosis factors for HCC. In addition, these genes appeared to be strongly associated with immune cell infiltration in HCC.

Presently, 276 DEGs were identified in three datasets. WGCNA was used to establish a co-expression network and reveal a turquoise module comprising genes that are significantly associated with clinical features of HCC patients. Univariate Cox regression was used to confirm the top 10 genes with low *P*-values in this module in the ICGC cohort. These genes were BIRC5, FOXM1, CENPA, KIF4A, DTYMK, PRC1, IGF2BP3, KIF2C, TRIP13, and TPX2. Survival curves and time-dependent ROC and AUC analyses indicated that the 10 hub genes have powerful predictive capacity for HCC. Most of the genes were validated in the GEPIA databases, except IGF2BP3. The univariate and multivariate Cox regression analyses of the hub genes showed that the genes were all independent predictors of HCC. The 10 genes were also confirmed to be associated with immune cell infiltration using the TIMER algorithm while we analyzed the methylation and mutation of the hub gene. On this basis, we constructed a risk score model and nomogram for prognostic prediction. Furthermore, the AUCs of the four-gene signature for 0.5-, 1-, 2-, 3-, and 5-year OS prediction models were 0.722, 0.793, 0.790, 0.819, and 0.800, indicating that the model had an excellent predictive capacity.

The 10 hub genes have been correlated with clinical outcomes of a huge number of solid tumors, especially HCC. BIRC5 promotes the progression of several gastrointestinal tumors, including HCC (Wheatley and Altieri, 2019). BIRC5 also promotes cell proliferation and invasion and inhibits apoptosis and cycle arrest (Su, 2016), and the aberrant methylation of BIRC5 was consistent basically with the previous report, which was identified by bioinformatics analysis (Cai et al., 2019). FOXM1 contributes to multiple cancers by promoting cellular proliferation and tumor initiation *via* β-catenin and cyclin D1 (Kim et al., 2019; Shukla et al., 2019). Bioinformatics analysis showed that FOXM1 was also involved in the development of hepatitis B virus (HBV)-related HCC (Xie et al., 2019). Aberrant CENPA expression participates in multiple stages of cancer progression by regulating the cell cycle (Sun et al., 2016). CENPA expression was reported to be significantly elevated in HCC tissues compared with normal tissues in TCGA and GEO, and the overexpression of CENPA was closely associated with HBV x gene (HBx) COOH mutation in HCC (Liu et al., 2012; Long et al., 2018). Gene concentration analysis revealed the pathway related to cell cycle and the p53 signal pathways as the most important pathways in the high-expression group of KIF4A in HCC, indicating that KIF4A plays a potential role in mediating the occurrence and development of tumors (Hou et al., 2017). DTYMK is a novel gene associated with mitochondrial DNA depletion syndrome (Lam et al., 2019) and prognosis of HCC (Yeh et al., 2017). BRC1 was reported to be a potential prognostic biomarker in various tumors, such as adrenocortical carcinoma (Xu W. H. et al., 2019) and non-muscle invasive bladder cancer (Shi et al., 2019). IGF2BP3 is a prognostic marker of poor outcome for colorectal cancer (Xu W. et al., 2019), glioma (Gao Q. et al., 2019; Zhang et al., 2019), and papillary renal cell carcinoma (Gao Z. et al., 2019). The overexpression of KIF2C has been significantly associated with poor prognosis of HCC (Chen et al., 2017). TRIP13 is overexpressed in HCC tissues and can induce progression and invasion of HCC (Yao et al., 2018; Zhu et al., 2019). The overexpression of KIF4A was suggested to promote the progression of HCC (Bai et al., 2019).

There are some limitations in this study. Our analysis was based on public data, and these datasets have been reported by other researchers. However, in this study, we analyzed the DEGs from GEO and ICGC and found out the co-expression module and key genes using WGCNA. Furthermore, we performed a multi-omics analysis for these key genes. Finally, we developed a four-gene signature and nomogram.

In summary, we screened 10 genes with marked prognostic capability for HCC. These genes were correlated with the infiltration of immune cells in HCC patients. The signaling pathways of these genes are involved in HCC. Importantly, we further determined that these hub genes are independent prognostic factors associated with OS of HCC patients. Moreover, we constructed a four-gene model, and the model was validated in TCGA. The findings might provide a new perspective that will further the understanding of the occurrence and development of HCC.

## Data Availability Statement

The GSE14520 and GSE22058 dataset were collected from the GEO with additional datasets obtained from the TCGA (https://portal.gdc.cancer.gov/) and ICGC (https://icgc.org/).

## Author Contributions

DW, JL, and WL designed the experiments and interpreted the data. SL, JL, and DW conducted bioinformatics and statistical analyses. DW and JL wrote the manuscript. All authors have read and approved the manuscript for publication.

## Conflict of Interest

The authors declare that the research was conducted in the absence of any commercial or financial relationships that could be construed as a potential conflict of interest.

## Footnotes

1 https://www.ncbi.nlm.nih.gov/geo/

2 https://icgc.org/

3 https://portal.gdc.cancer.gov/

4 https://bioinformatics.psb.ugent.be/webtools/Venn/

5 www.genome.jp/kegg/pathway.html

6 http://bioinfo.life.hust.edu.cn/web/GSCALite/

7 http://gepia.cancer-pku.cn/

8 http://ualcan.path.uab.edu/index.html

9 https://www.cbioportal.org/

10 https://cistrome.shinyapps.io/timer/

## Supplementary Material

FIGURE S1 Volcano map of differentially expressed genes. Differentially expressed genes in GSE14520 **(A)**, GSE22058 **(B)**, ICGC-JP **(C)**. Green represented down-regulation genes, red represented up-regulation, black represented non-significantly differentially genes.

FIGURE S2 Gene correlation analysis in the module. Expression of BIRC5 **(A)**, FOXM1 **(B)**, CENPA **(C)**, KIF4A **(D)**, DTYMK **(E)**, PRC1 **(F)**, IGF2BP3 **(G)**, KIF2C **(H)**, TRIP13 **(I)**, and TPX2 **(J)** associated with the turquoise module.

FIGURE S3 The expression levels of hub genes among different clinical stages in the ICGC cohort, including BIRC5 **(A)**, FOXM1 **(B)**, CENPA **(C)**, KIF4A **(D)**, DTYMK **(E)**, PRC1 **(F)**, IGF2BP3 **(G)**, KIF2C **(H)**, TRIP13 **(I)**, and TPX2 **(J)**.

FIGURE S4 The expression levels of hub genes among different clinical stages in GEPIA dataset, including BIRC5 **(A)**, FOXM1 **(B)**, CENPA **(C)**, KIF4A **(D)**, DTYMK **(E)**, PRC1 **(F)**, IGF2BP3 **(G)**, KIF2C **(H)**, TRIP13 **(I)**, and TPX2 **(J)**.

FIGURE S5 The survival analysis of 10 hub genes in GEPIA database, including BIRC5 **(A)**, FOXM1 **(B)**, CENPA **(C)**, KIF4A **(D)**, DTYMK **(E)**, PRC1 **(F)**, IGF2BP3 **(G)**, KIF2C **(H)**, TRIP13 **(I)**, and TPX2 **(J)**.

FIGURE S6 The hub gene expressions correlated with macrophage polarization in HCC, including BIRC5 **(A)**, FOXM1 **(B)**, CENPA **(C)**, KIF4A **(D)**, DTYMK **(E)**. Markers include purity, B cell, CD8^+^ T cell, CD4^+^ T cell, macrophages, neutrophil, and dendritic cell.

FIGURE S7 The hub gene expressions correlated with macrophage polarization in HCC, including PRC1 **(A)**, IGF2BP3 **(B)**, KIF2C **(C)**, TRIP13 **(D)**, and TPX2 **(E)**. Markers include purity, B cell, CD8^+^ T cell, CD4^+^ T cell, macrophages, neutrophil, and dendritic cell.

FIGURE S8 The hub genes express in HCC. The expression profiles of the four genes in TCGA liver cancer RNA-seq (*n* = 371) dataset.

FIGURE S9 Stratification of patients based on the median risk score. The distribution of risk score (upper), survival time (middle) and mRNA expression (below) in ICGC **(A)** and TCGA **(B)**.
