# Supplementary figures and images for: Identification of Crucial Genes Associated With Immune Cell Infiltration in Hepatocellular Carcinoma by Weighted Gene Co-expression Network Analysis
Source: Front Genet. 2020 Apr 24;11:342. doi: 10.3389/fgene.2020.00342 (PMC7193721; doi:10.3389/fgene.2020.00342)

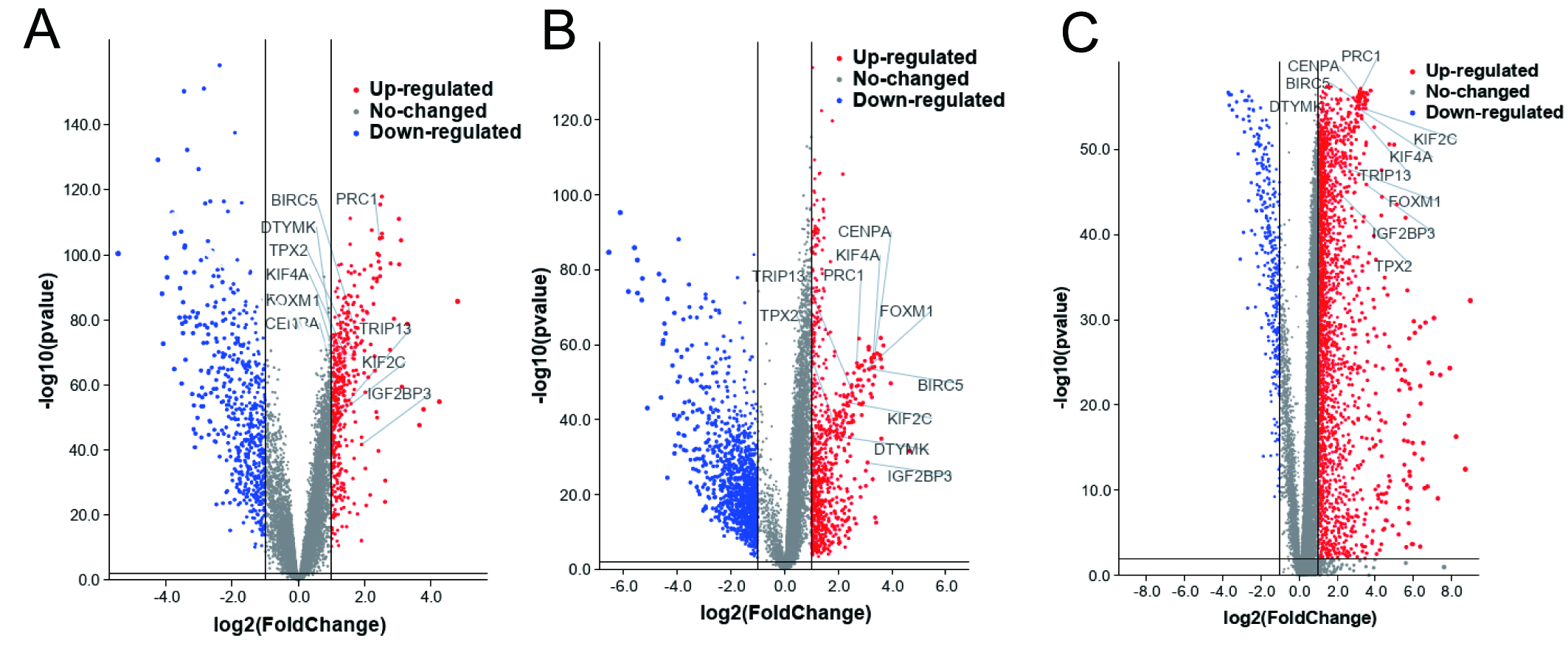

Supplement: FIGURE S1 — Volcano map of differentially expressed genes. Differentially expressed genes in GSE14520 (A), GSE22058 (B), ICGC-JP (C). Green represented down-regulation genes, red represented up-regulation, black represented non-significantly differentially genes. [file Image_1.TIF]

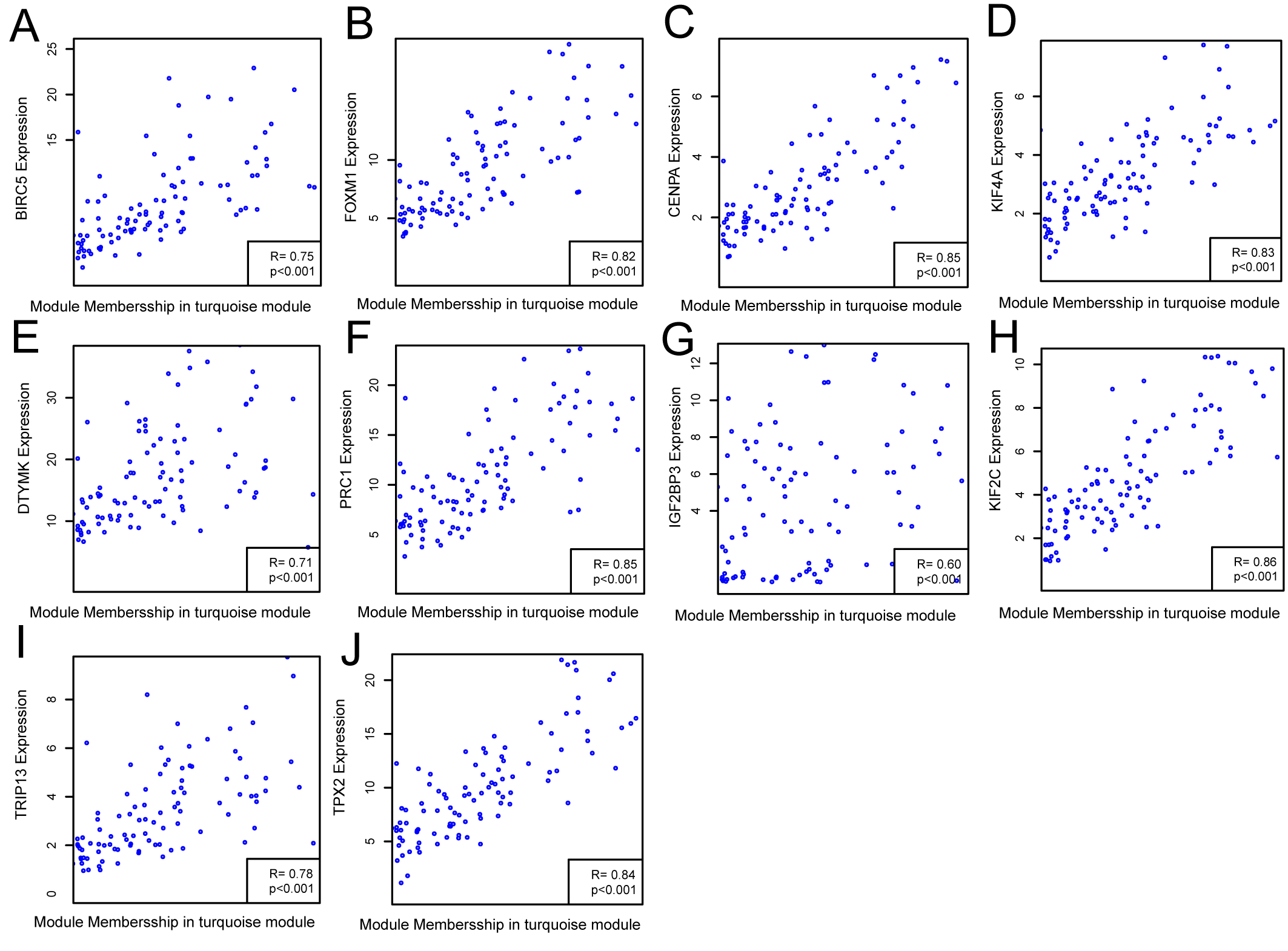

Supplement: FIGURE S2 — Gene correlation analysis in the module. Expression of BIRC5 (A), FOXM1 (B), CENPA (C), KIF4A (D), DTYMK (E), PRC1 (F), IGF2BP3 (G), KIF2C (H), TRIP13 (I), and TPX2 (J) associated with the turquoise module. [file Image_2.TIF]

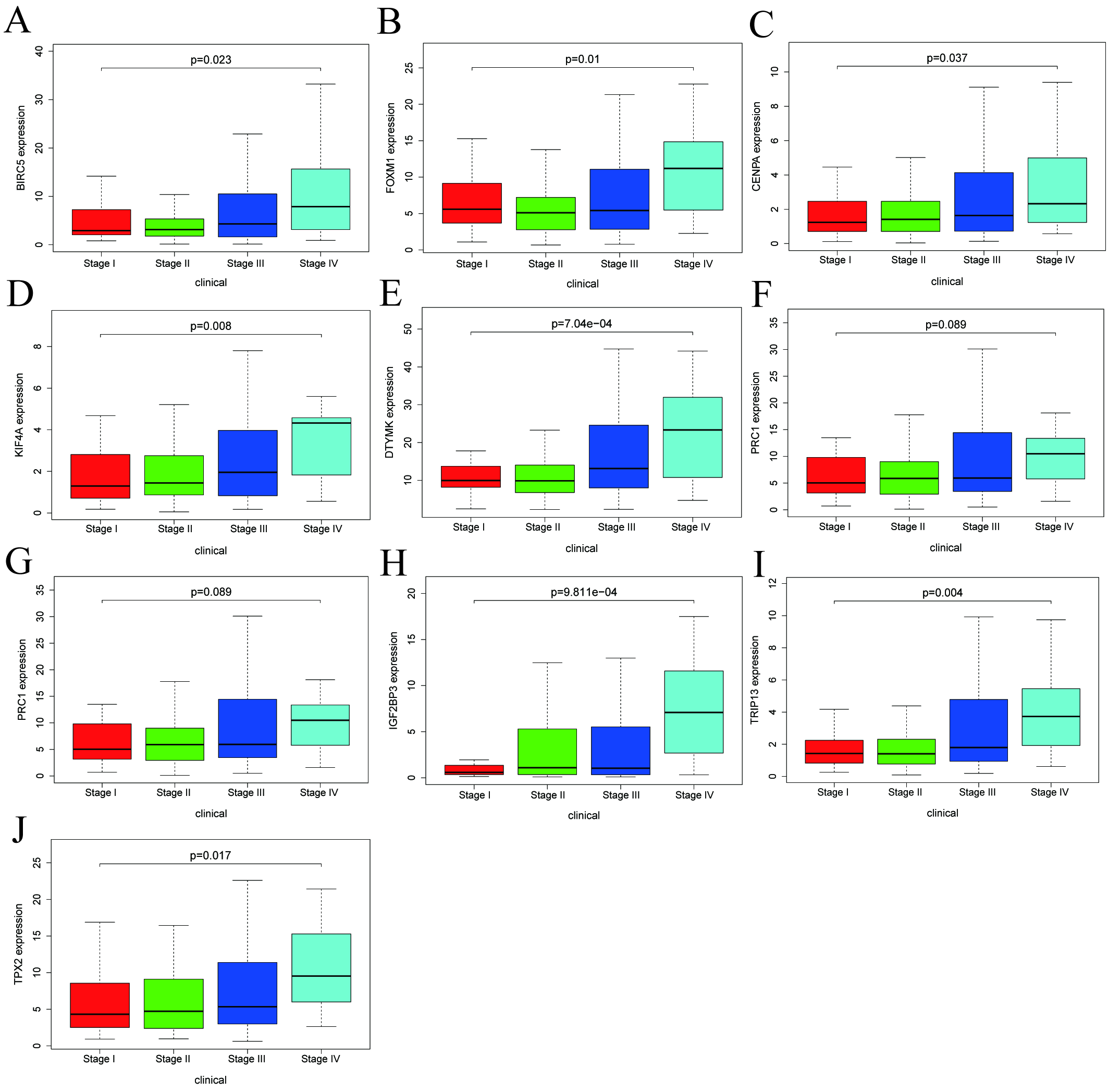

Supplement: FIGURE S3 — The expression levels of hub genes among different clinical stages in the ICGC cohort, including BIRC5 (A), FOXM1 (B), CENPA (C), KIF4A (D), DTYMK (E), PRC1 (F), IGF2BP3 (G), KIF2C (H), TRIP13 (I), and TPX2 (J). [file Image_3.TIF]

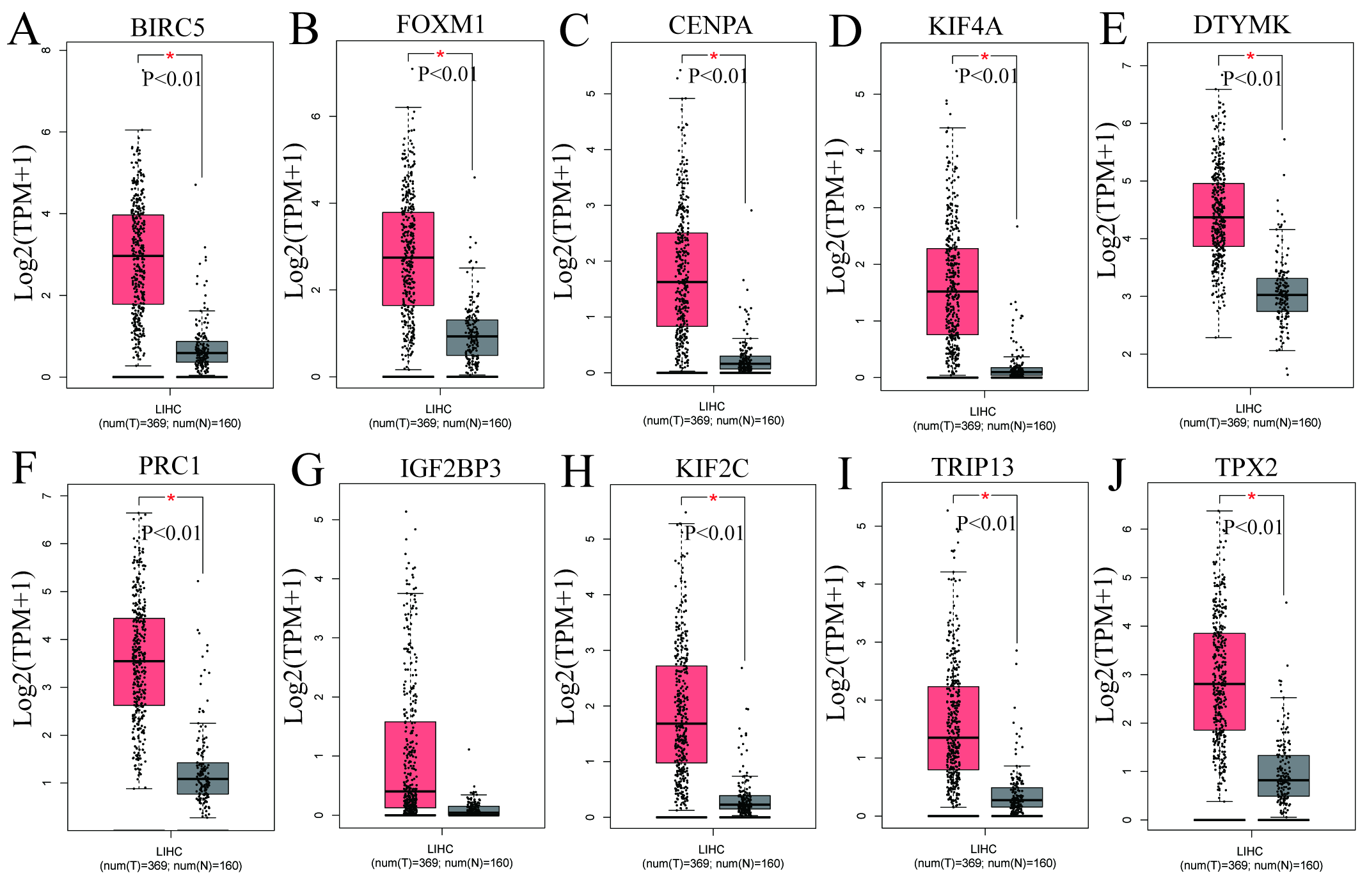

Supplement: FIGURE S4 — The expression levels of hub genes among different clinical stages in GEPIA dataset, including BIRC5 (A), FOXM1 (B), CENPA (C), KIF4A (D), DTYMK (E), PRC1 (F), IGF2BP3 (G), KIF2C (H), TRIP13 (I), and TPX2 (J). [file Image_4.TIF]

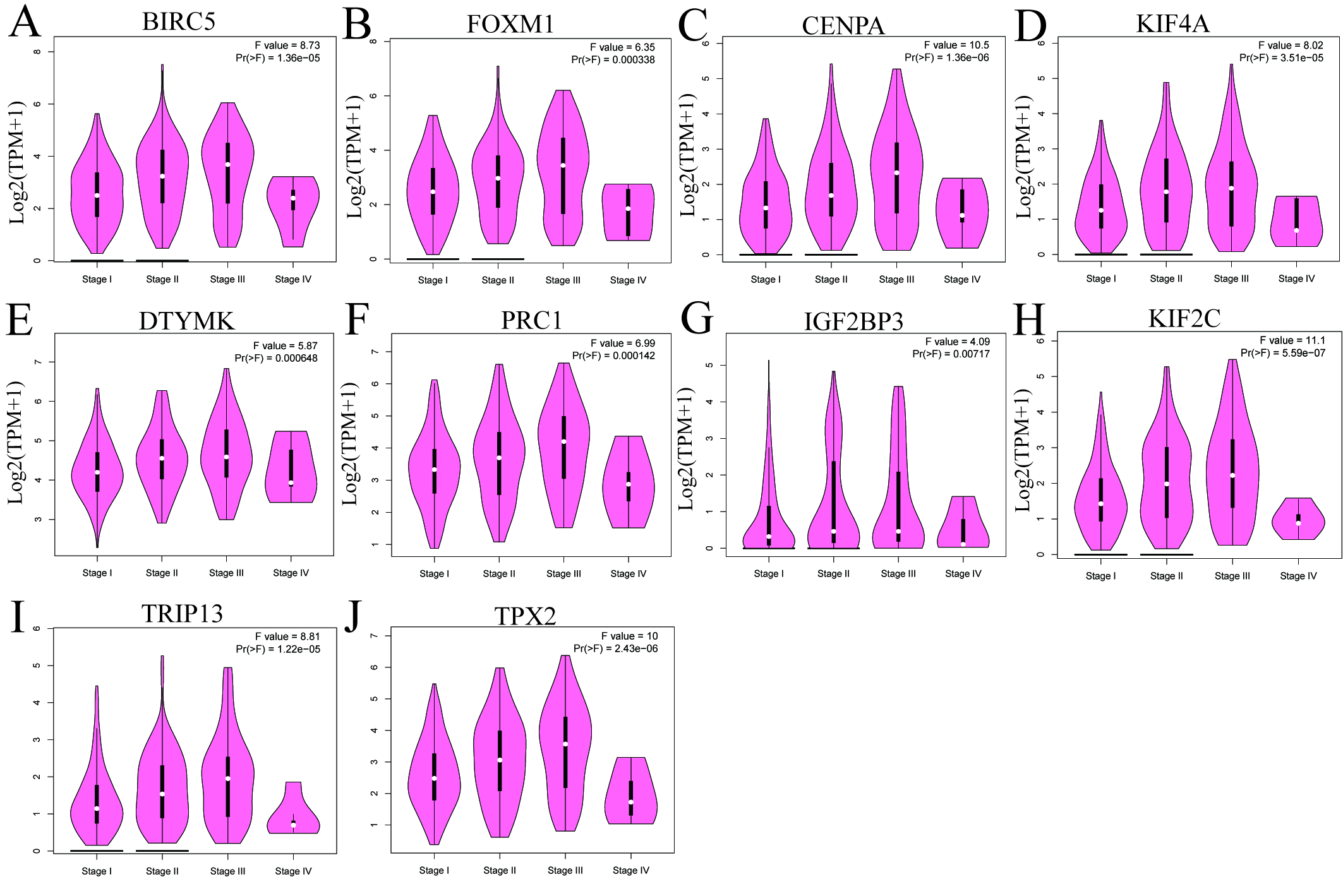

Supplement: FIGURE S5 — The survival analysis of 10 hub genes in GEPIA database, including BIRC5 (A), FOXM1 (B), CENPA (C), KIF4A (D), DTYMK (E), PRC1 (F), IGF2BP3 (G), KIF2C (H), TRIP13 (I), and TPX2 (J). [file Image_5.TIF]

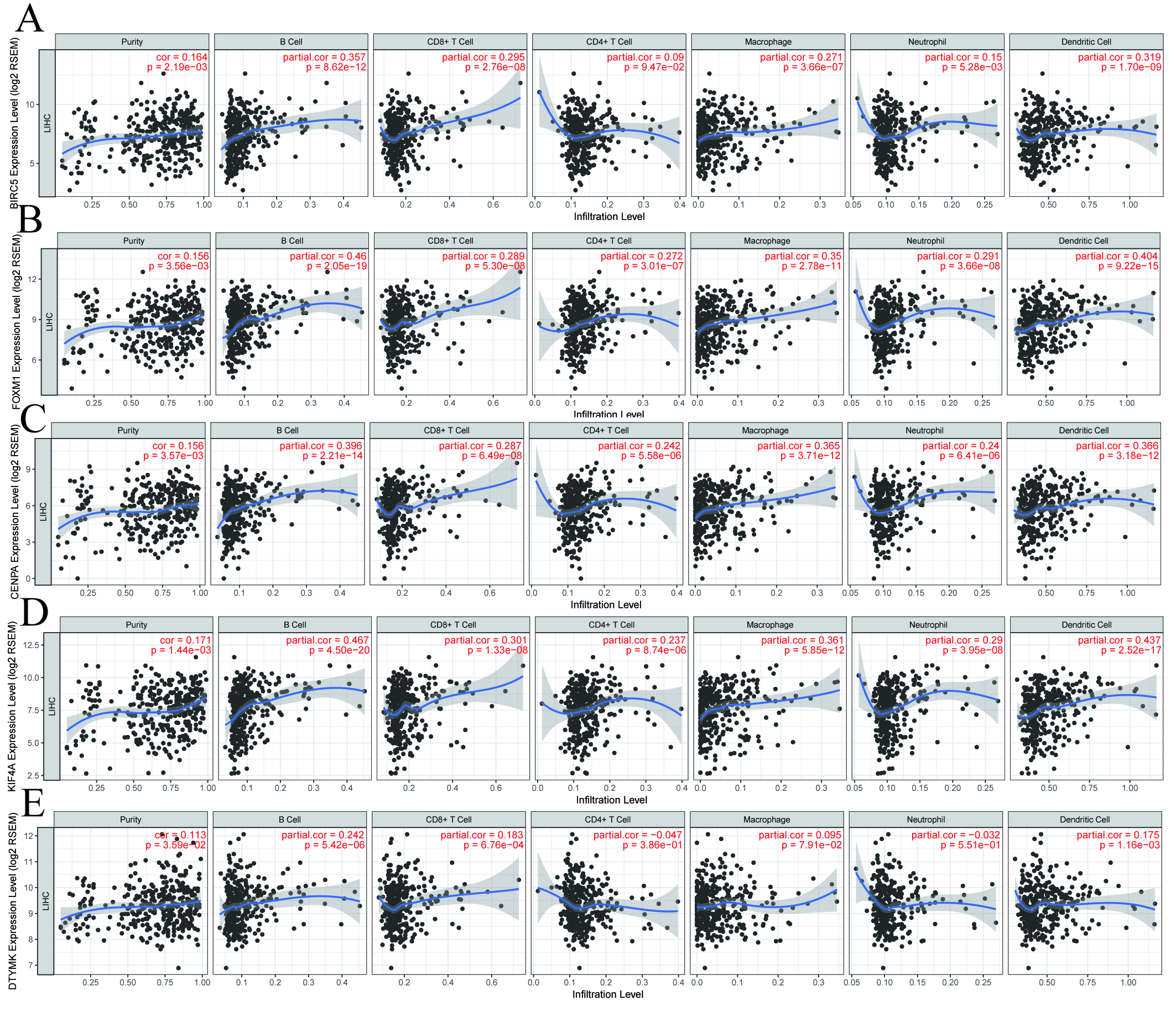

Supplement: FIGURE S6 — The hub gene expressions correlated with macrophage polarization in HCC, including BIRC5 (A), FOXM1 (B), CENPA (C), KIF4A (D), DTYMK (E). Markers include purity, B cell, CD8+ T cell, CD4+ T cell, macrophages, neutrophil, and dendritic cell. [file Image_6.TIF]

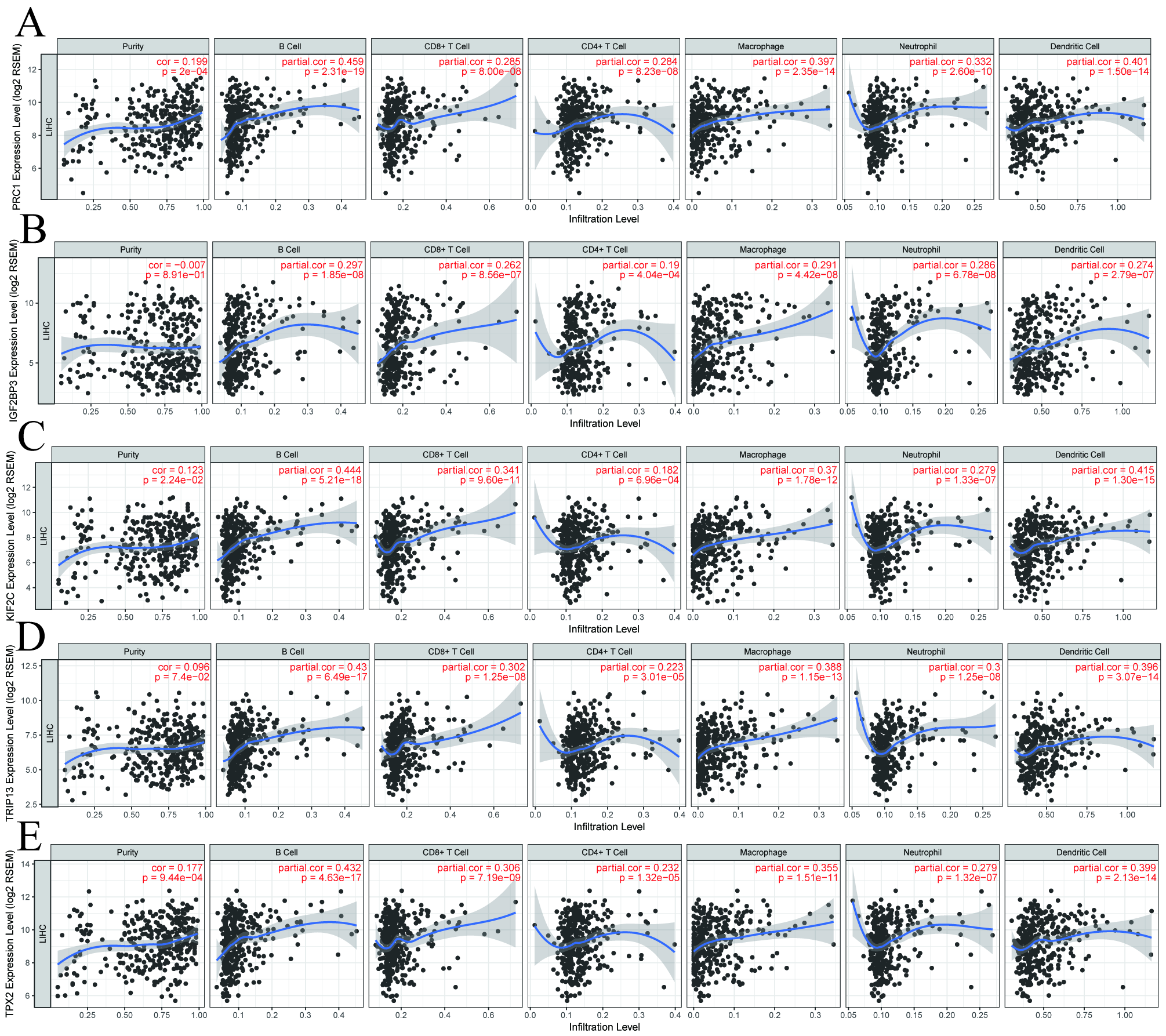

Supplement: FIGURE S7 — The hub gene expressions correlated with macrophage polarization in HCC, including PRC1 (A), IGF2BP3 (B), KIF2C (C), TRIP13 (D), and TPX2 (E). Markers include purity, B cell, CD8+ T cell, CD4+ T cell, macrophages, neutrophil, and dendritic cell. [file Image_7.TIF]

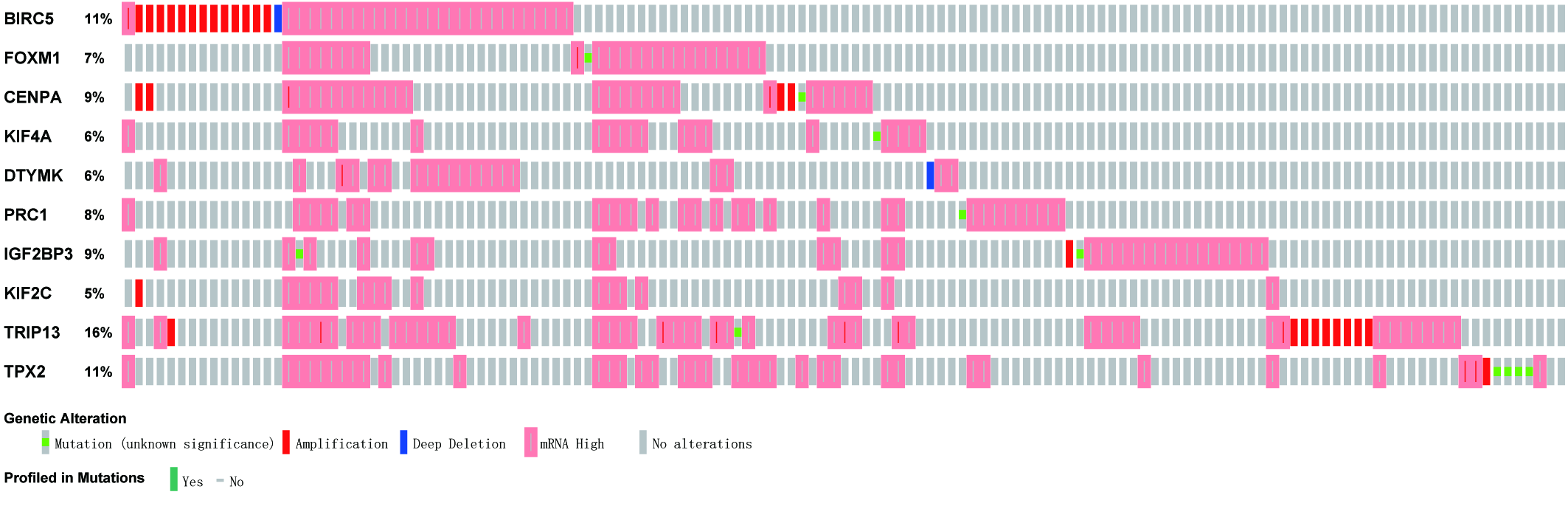

Supplement: FIGURE S8 — The hub genes express in HCC. The expression profiles of the four genes in TCGA liver cancer RNA-seq (n = 371) dataset. [file Image_8.TIF]

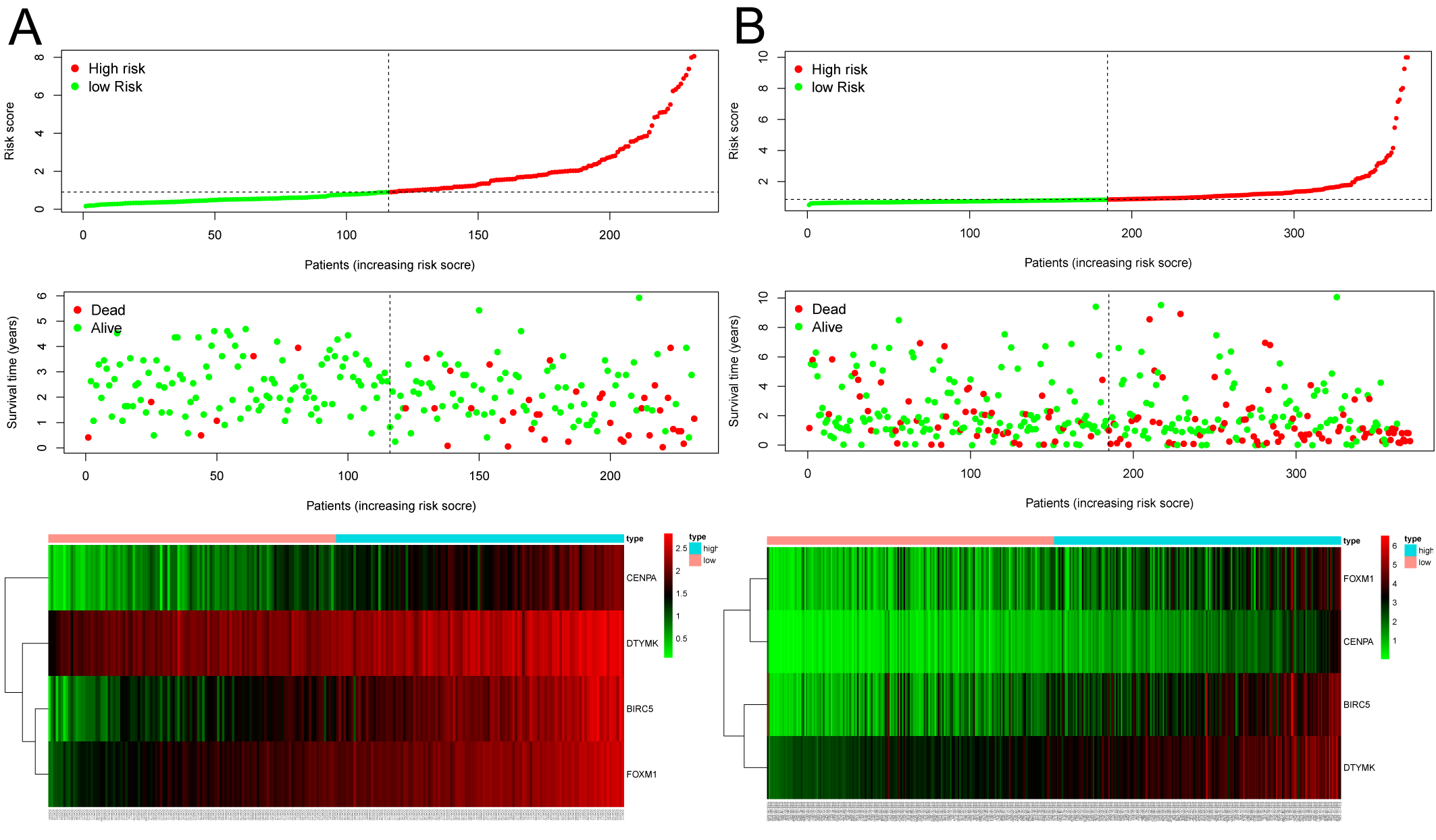

Supplement: FIGURE S9 — Stratification of patients based on the median risk score. The distribution of risk score (upper), survival time (middle) and mRNA expression (below) in ICGC (A) and TCGA (B). [file Image_9.TIF]
